# On-Chip Impedance Spectroscopy of Malaria-Infected Red Blood Cells

**DOI:** 10.3390/s24103186

**Published:** 2024-05-17

**Authors:** Nitipong Panklang, Boonchai Techaumnat, Nutthaphong Tanthanuch, Kesinee Chotivanich, Mati Horprathum, Michihiko Nakano

**Affiliations:** 1 Department of Electrical Engineering, Faculty of Engineering, Rajamangala University of Technology Thanyaburi, Pathum Thani 12110, Thailand; nitipong.p@en.rmutt.ac.th; 2 Department of Electrical Engineering, Faculty of Engineering, Chulalongkorn University, Bangkok 10330, Thailand; 3 Micro/Nano-Electro-Mechanical Integrated System Research Unit, Faculty of Engineering, Chulalongkorn University, Bangkok 10330, Thailand; 4 Department of Electrical and Computer Engineering, Faculty of Engineering, Thammasat School of Engineering, Thammasat University, Pathum Thani 12120, Thailand; tanthanuch1@engr.tu.ac.th; 5 Cell and Tissue Culture Resources Unit, Department of Clinical Tropical Medicine, Faculty of Tropical Medicine, Mahidol University, Bangkok 10400, Thailand; nok@tropmedres.ac; 6 Spectroscopic and Sensing Devices Research Group, NECTEC, NSTDA, Pathum Thani 12120, Thailand; mati.horprathum@nectec.or.th; 7 Faculty of Information Science and Electrical Engineering, Kyushu University, Fukuoka 819-0395, Japan; nakano@ees.kyushu-u.ac.jp

**Keywords:** electrical impedance spectroscopy, microfluidic device, malaria-infected cell, red blood cell, cell orientation

## Abstract

Malaria is a disease that affects millions of people worldwide, particularly in developing countries. The development of accurate and efficient methods for the detection of malaria-infected cells is crucial for effective disease management and control. This paper presents the electrical impedance spectroscopy (EIS) of normal and malaria-infected red blood cells. An EIS microfluidic device, comprising a microchannel and a pair of coplanar electrodes, was fabricated for single-cell measurements in a continuous manner. Based on the EIS results, the aim of this work is to discriminate *Plasmodium falciparum*-infected red blood cells from the normal ones. Different from typical impedance spectroscopy, our measurement was performed for the cells in a low-conductivity medium in a frequency range between 50 kHz and 800 kHz. Numerical simulation was utilized to study the suitability parameters of the microchannel and electrodes for the EIS experiment over the measurement frequencies. The measurement results have shown that by using the low-conductivity medium, we could focus on the change in the conductance caused by the presence of a cell in the sensing electrode gap. The results indicated a distinct frequency spectrum of the conductance between the normal and infected red blood cells, which can be further used for the detection of the disease.

## 1. Introduction

Malaria is an infectious disease that is caused by a Plasmodium parasite. The malarial parasite infects humans and other animals through the bites of infected female mosquitoes. For human malaria, there are seven different species of *Plasmodium* parasite, namely *P. falciparum*, *P. vivax*, *P. malariae*, *P. ovale curtisi*, *P. ovale wallikeri*, *P. knowlesi*, and *P. cynomolgi* [1]. The ongoing increase in global average temperature due to climate change has a direct impact on several infectious diseases including malaria. Environmental change makes it easier for mosquitoes, which are the intermediate host of malaria, to spread their habitats. It expands the range of malaria transmission to many areas where patients have not been previously found [2,3]. The expansion of malaria transmission requires early detection for the appropriate treatment. In 2021, approximately 247 million cases of malaria are reported representing a 2 million case increase from the previous year. In addition, global funding for malaria control and elimination has shown an increasing trend with USD 3.5 billion allocated in 2021, up from USD 3.3 billion in 2020 and USD 3.0 billion in 2019 [4].

By malarial infection, the parasite affects various properties of a red blood cell (RBC). For example, there are changes in intracellular media, reduction of deformability, and increasing adhesiveness of RBC to other cells and to blood vessels [5,6]. Detection of the change in cellular properties of the malaria-infected cells is, thus, an essential means for improving the precision and accuracy of malaria diagnosis. In addition, the electrical cellular properties of the infected cells can also be used as an index for developing effective treatments.

For the inspection of the intracellular properties of biological cells, there are several methods such as dielectrophoresis (DEP) [7,8,9,10,11,12], electrorotation (EROT) [13,14,15], and electrical impedance spectroscopy (EIS) [16,17,18]. DEP and EROT are indirect electromechanical methods by which the linear and angular motion of cells under an externally applied electric field are observed, respectively. The electrical cellular properties can be analyzed from the critical frequencies, at which the cells show a minimal or maximal degree of movement. The methods give clear information about the critical frequencies, which reflect changes in cellular characteristics, and they are less dependent on electrical noises and geometrical parameters of the measurement platform. However, the DEP and EROT operation for high throughput is difficult to realize due to the nature of the methods as the critical frequencies of single cells are typically determined from the observation of cell motions under a variation of field frequency [19].

EIS is an electrical method based on a voltage-current measurement to estimate the electrical properties of targets such as biological cells or particles located between activating and sensing electrodes. EIS was used to study the electrical properties of biological cells such as cancer cells [20,21], yeast cells [22,23,24], and bacteria [25,26]. The EIS has the same advantages as the DEP and EROT, in that it is a label-free and non-invasive technique that does not require cell modifications such as dyeing, staining, or labeling. For single-cell EIS, a microchannel is typically employed for the measurement. As electrical responses can be obtained faster than electromechanical ones, the EIS presents an alternative to achieve higher throughput for examining changes in cellular properties [27]. Microfluidic devices were employed to investigate the cellular properties of blood cells [28,29,30,31]. The use of EIS for identifying normal red blood cells from latex beads and ghost blood cells was reported [32]. A microfluidic device and coplanar electrodes were used to measure the impedance of the particles and the cells diluted in standard PBS. The proposed device can discriminate between different cell types. However, the sensing area of the device was rather wide, which might not suit a single-cell inspection. The application of EIS for characterizing late-stage *P. falciparum* was carried out using a modified surface gold electrode [33]. The surface was modified by depositing antigen/antibody for immobilization of cells during the measurement, which complicated the fabrication and preparation. The properties of malaria-infected cells in various stages were investigated using an EIS-based device combined with fluorescence microscopy [34]. Electrode alignment on opposite sides of the microchannel was needed. Using double-shelled cell models, a significant change in the membrane permittivity and cytoplasmic conductivity were demonstrated. A miniature device based on EIS was used to distinguish between P. falciparum-infected cells and normal blood cells [35]. It employed a near-field communication (NFC) interface to visualize the impedance spectrum. However, the detection volume was large, possibly affecting the resolution of the results. A portable biosensor system that utilizes EIS for the detection of malaria parasites was demonstrated [36]; however, the experiments used only fluorescent beads of different sizes representing healthy and infected red blood cells.

In this work, we present the electrical impedance spectroscopy of normal red blood cells (nRBCs) and malaria-infected red blood cells (iRBCs). The EIS experiments for nRBCs and iRBCs were conducted using a simple microfluidic device. The device consisted of a microchannel with coplanar electrodes for the sake of fabrication and operation simplicity. Our objective is to differentiate between iRBCs and nRBCs based on the obtained impedance spectrum. Different from the existing works, we studied the electrical characteristics of the cells in a medium of low conductivity. The EIS measurement was performed in a single-cell, continuous process, allowing throughput enhancement. Numerical simulations were utilized to determine the suitability of the microchannel and electrode dimensions for EIS measurements and to estimate detectable changes in the impedance values due to cellular properties over the frequencies of measurement. The utilization of the low-conductivity medium permitted the classification of infected cells directly by the change in the conductance-frequency characteristic. The results obtained here can be extended to realize an on-chip malaria diagnosis. In addition, it is ready to be used for evaluating the efficiency of DEP-based cell sorting, which is typically performed in low-conductivity media.

## 2. Theory

**Figure 1:**
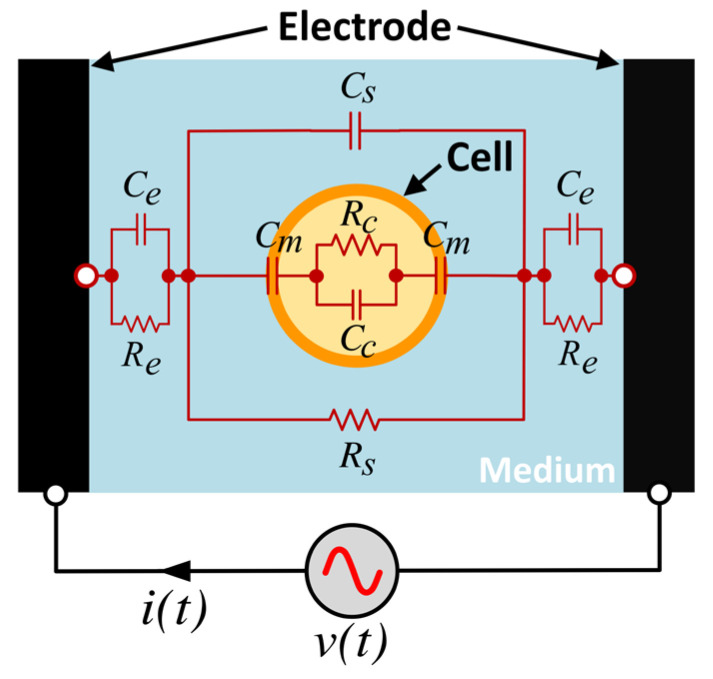
Approximate equivalent circuit of cell in a suspending medium between electrodes.

Electrical impedance spectroscopy (EIS) is a non-invasive method for examining the electrical properties of biological cells as a function of the electrical frequency. Based on a viewpoint of the electrical circuit, the fundamental concept of EIS is shown in Figure 1. Consider a biological cell suspended in a medium in between a pair of sensing electrodes. The system of the cell and the extracellular medium can be represented approximately by an equivalent circuit consisting of resistors and capacitors. In the equivalent circuit, $C_{m}$ is the cell membrane capacitance, $R_{c}$ and $C_{c}$ are the intracellular resistance and capacitance, respectively. The resistance and capacitance of the circuit parallel to the cell (in the suspending medium) are denoted by $R_{s}$ and $C_{s}$, respectively. $R_{e}$ and $C_{e}$ are the resistance and capacitance of the bulk separated from the cell including those at the interface between electrodes and the suspending medium.

Let a sinusoidal applied voltage *v*(*t*) of frequency *f* be

(1) $$v\left(t\right)=V_{m}sin(2\pi ft),$$

and current i(t) between electrodes be

(2) $$i\left(t\right)=I_{m}sin(2\pi ft+\theta ),$$

where $V_{m}$ and $I_{m}$ are the voltage and current amplitudes, respectively, and *θ* is the phase difference between the voltage and the current. The admittance **Y** between the sensing electrodes is

(3) Y=I/V=G+jB,

where V and I are phasors of v(t) and i(t), respectively. *G* and *B* are the conductance and susceptance between the electrodes, respectively, and $j=\sqrt{-1}$.

For resistor–capacitor (RC) circuits, the admittance between the sensing electrodes varies with frequency *f*. At intermediate frequencies, the main factor for dominating the admittance is the interfacial polarization between the cell and the medium. Therefore, they are usually applied to the EIS to investigate the cell size and the cell membrane properties. At higher frequencies, the cell membrane capacitance is essentially bypassed, and the properties of the cytoplasm are reflected by the measured admittance [17].

## 3. Materials and Methods

### 3.1. Cell Sample

Normal red blood cells (nRBCs) and malaria-infected red blood cells (iRBCs) were used for the EIS measurement. Cell samples were cultured by the cell and tissue culture resources unit (CTCRU), Faculty of Tropical Medicine, Mahidol University. The culture medium was RPMI-1640 containing HEPES, NaHCO_3_, gentamicin 40 mg/mL, and 10% (*v*/*v*) heat-inactivated human AB serum. For the malaria infection, the suspension of red blood cells was incubated with *Plasmodium falciparum* for 30–36 h at 37 °C and 5% CO_2_. During the incubation period, we obtained infected cells in either trophozoite or schizont stages. The infected cells were then purified using a magnetic column (LS Column, Miltenyi Biotec, Bergisch Gladbach, Germany) and resuspended in RPMI-1640. A low-conductivity medium was made from 248 mM Sucrose and 16.65 mM Dextrose in deionized water (DI). Bovine serum albumin (BSA) was added to reduce the adhesion of cells. The conductivity of the medium was adjusted to 0.02 S/m using a phosphate buffered saline (PBS). Before the experiment, the cell samples were washed three times by centrifugation at 1500× *g* rpm for 5 min with the low-conductivity medium. Then, 2 µL of the cell suspension was added to 1000 µL of the low-conductivity medium for the EIS measurement. The cell density was about 2 × 10^4^ cells/µL. The value was determined experimentally to avoid clogging and to obtain appropriate space between cells for the measurement by our equipment. The cell suspension was kept below 10 °C during the experiments.

### 3.2. Device

**Figure 2:**
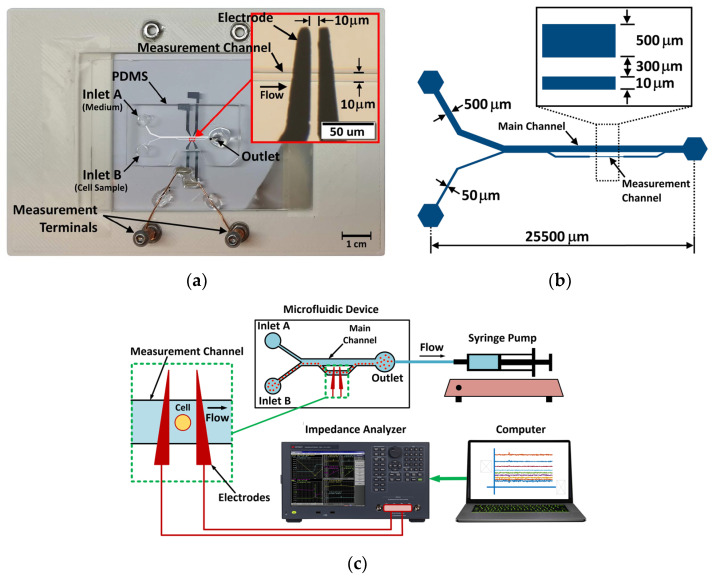
Microfluidic device for EIS experiment. (**a**) Actual device, (**b**) dimensions of the microchannel, and (**c**) schematic diagram of the EIS measurement setup.

The microfluidic device used for the measurement is shown in Figure 2a. The microchannel consisted of a main channel of 500 µm width and a measurement channel of 10 µm width as shown in Figure 2b. The depth of the microchannel was 8.5 µm. The device had two inlets for feeding a cell suspension and a blank (cell-free) low-conductivity solution into the device. The sheath flow of the blank solution in the main channel confined the red blood cells in the sample to the measurement channel. A pair of coplanar electrodes were set in the measurement channel for sensing electrical impedance. The electrodes had an average width of 15 µm and the gap between them was 10 µm.

The mold of the microchannel was made on a glass substrate with a photoresist (SU-8 5, MicroChem, Austin, TX, USA) using a photolithography process. The photoresist was coated using a spin coater with a speed of approximately 1900 rpm. After UV exposure, the photoresist was developed and then hard-baked at 120 °C for 5 min. The microchannel was made of polydimethylsiloxanes (PDMS, KE-106, ShinEtsu, Tokyo, Japan) which were mixed with the susceptance catalyst (CAT-RG, ShinEtsu, Tokyo, Japan) at a ratio of 10:1. The PDMS was poured on the mold and cured at 95 °C for 1 h on hotplate. The microchannel consisted of main and measurement channels. Figure 2b illustrates the dimensional details of the microchannel.

The coplanar electrodes were fabricated using a liftoff process. The electrode pattern was made on a glass substrate with a negative photoresist (NR9-3000PY, Futurrex, Franklin, NJ, USA). Chromium was deposited on the substrate using a sputtering process. Then, the photoresist was removed to obtain the patterned microelectrodes. The thickness of the electrodes was approximately 200 nm. The microfluidic device was set on a plastic base, and conductor wires connected the electrodes to terminals for electrical measurement. The inset of Figure 2a shows the electrode placement in the measurement channel. 

### 3.3. Experimental Setup

Figure 2c shows the schematic diagram of the EIS measurement setup. Before feeding a cell sample, the microchannel was coated with a solution of bovine serum albumin (HiMEDIA, Thane, India) in deionized water. The cell suspension was fed into the microfluidic device through inlet B, while the medium without cells was fed into inlet A. The outlet of the device was connected to a syringe pump (Fusion 200, Chemyx, Stafford, TX, USA) to draw the cell sample and the blank solution into the device at a flow rate of 0.003 µL/min. Cells were forced to flow through the measurement channel by the sheath flow. The impedance between the electrodes was measured by an impedance analyzer (E4990A, Keysight, Santa Rosa, CA, USA). The impedance analyzer was controlled by an in-house MATLAB program, and the measured data were sent to a computer via a universal serial bus (USB) connection for subsequent analysis.

### 3.4. Numerical Simulation

**Figure 3:**
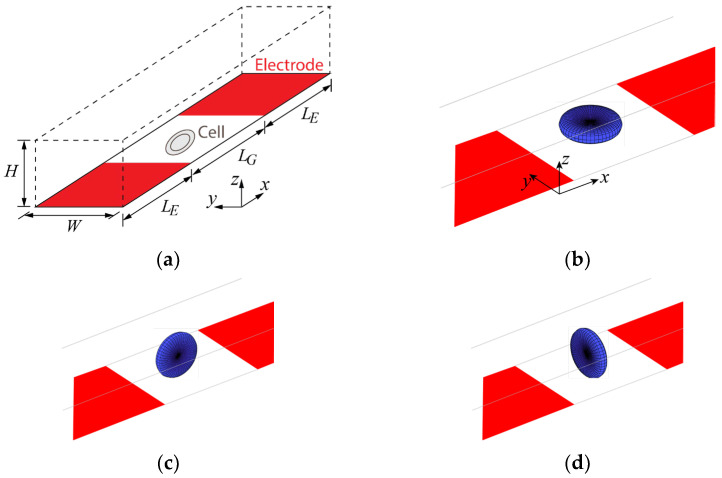
Numerical simulation. (**a**) Configuration, (**b**–**d**) different cell orientations treated in the simulation.

Numerical simulation has been applied to determine whether the channel and electrode dimensions are suitable for the EIS measurement of a single red blood cell. Figure 3a shows the configuration that was used in the simulation. A red blood cell is located in a gap between two planar electrodes in the microchannel of width *W* and height *H*. The coplanar electrodes of length *L_E_* and gap *L_G_* are set on the bottom of the microchannel. The red blood cell was modeled as a biconcave disc, having a radius of 4.2 μm and a maximum thickness of 2.04 μm, to investigate the effect of its orientation on the measurement results. The cell dimensions were referred from previous work [37]. Figure 3b–d shows the three orientations of the cell considered in this work. The circular face of the cell is parallel with the electrode plane in Figure 3b, but normal to the plane in Figure 3c,d. The circular face is approximately parallel and normal to electric field lines in Figure 3c,d, respectively.

The finite element method (FEM) was applied to the electric field calculation. The cell membrane was modeled by using zero-thickness elements. Neglecting membrane conductance for normal red blood cells, we applied the boundary condition of the cell membrane as

(4) $$(\sigma +j\omega \varepsilon )E_{n}=j\omega C_{m}V_{m},$$

where σ and ε were the conductance and the permittivity of the medium on either side of the membrane, respectively, $E_{n}$ was the normal component of the electric field, ω was the electric frequency, $C_{m}$ was the specific membrane capacitance, and $V_{m}$ was the transmembrane voltage. For the simulation, the intracellular permittivity $\varepsilon_{c}$ was 60$\varepsilon_{0}$, conductivity $\sigma_{c}$ was 0.328 S/m, and $C_{m}$ = 0.912 µF/cm^2^ [38]. The extracellular medium was an aqueous solution with a 0.02 S/m conductivity, referred from our experimental condition.

## 4. Results and Discussion

### 4.1. Simulation

**Figure 4:**
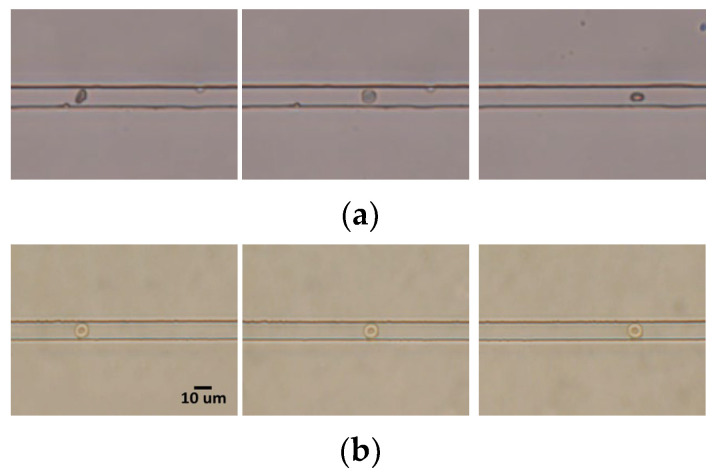
Images of red blood cells traveled in the measurement microchannel where the channel height was (**a**) 10 μm and (**b**) 8.5 μm.

As already mentioned, one of the main purposes of the simulation was to determine the influence of cell orientation and the propriety of microchannel and electrode dimensions. Numerical simulation for a specific frequency was applied to determine the positions of bioparticles in a microchannel [39]. In the current work, we computed the electrical response on a range of frequencies (*f*) from 10 kHz and 10 MHz to observe the Δ*G-f* characteristics. Δ*G* is defined as the difference between the conductance in the presence of a red blood cell in the sensing gap and that in the absence of the cell. We observed actual cell orientation in the microchannel with different heights (*H*), 10 μm and 8.5 μm, where the dimensions of *W*, *L_E_*, and *L_G_* were used as specified in Section 3.2. Figure 4 shows the images of cells while they moved through the measurement microchannel in a preliminary experiment. While a red blood cell traveled along the microchannel, the change in orientation of the cell could be observed, as shown in Figure 4a, where the channel height was 10 μm. The use of larger heights (*H*) relaxed the experimental conditions as it reduced the possibility of channel clogging. On the other hand, we found that the cells were kept in the orientation of Figure 3b with reducing *H* to 8.5 μm, as shown in Figure 4b.

**Figure 5:**
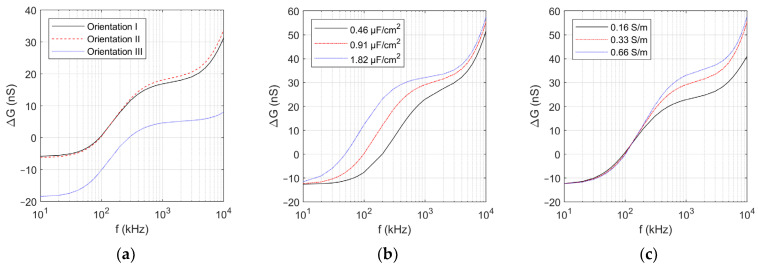
Variation of the conductance difference Δ*G* with (**a**) cell orientation, (**b**) cell membrane capacitance, and (**c**) intracellular conductivity.

Figure 5a compares the variation of Δ*G* as a function of electrical frequency (*f*) between the cell orientations. Note that orientations I, II, and III refer to those in Figure 3b–d, respectively. It is clear from Figure 5 that for all orientations, Δ*G* is negative at low frequencies, but changes to be positive at high frequencies. The effect of the orientation is also clearly seen in Figure 5a. The cell in orientation III of Figure 3d tends to yield smaller Δ*G* at all frequencies, compared with the other two orientations. It is worth noting that the critical frequency (*f_c_*) at which Δ*G* changes its sign also significantly varies with the orientation of the nonspherical cells. Therefore, based on the obtained simulation result, the height (*H*) = 8.5 μm was adopted for the EIS experiment so that we could restrict the cell orientation and attain more consistent measurement results. From the results, the sign of Δ*G* is to be used for cell classification.

Using orientation I of Figure 3b, we computed Δ*G* for the red blood cell having different values of membrane-specific capacitance and intracellular conductivity. Figure 5b,c shows the simulation results. As can be seen from Figure 5b, a decrease in the capacitance results in a higher critical frequency (*f_c_*) that lowers Δ*G* in the frequency range considered in this work. On the other hand, Figure 5c indicates that the variation of the intracellular conductivity has minimal effect on the *f_c_* value, but Δ*G* at the higher frequencies tends to increase with the intracellular conductivity. The simulation results implied that our EIS performed in the frequency range from 50 to kHz 800, and the change Δ*G* at the lower and intermediate frequencies of the range should reflect the change on the cell membrane while the upper frequencies implied the change in the intracellular property. Hence, the malarial infection, which begins with the invasion of the cell membrane by the parasite, is expected to be detectable in the applied frequency range. 

### 4.2. Experiment

**Figure 6:**
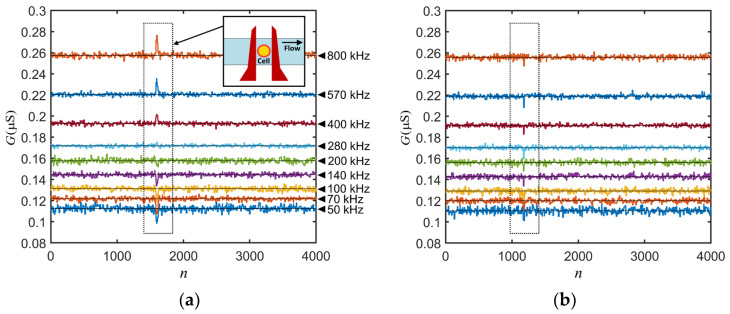
Conductance between electrodes for (**a**) a normal red blood cell and (**b**) an infected red blood cell.

We performed the experiment on normal red blood cells (nRBCs) and malaria-infected red blood cells (iRBCs). The experiments were performed with two sets of cell samples. The first one consisted of only normal cells for use as a reference. The second one comprised late-stage malaria-infected red blood cells with an approximate infection rate of 40%. Figure 6a,b shows an example of the measurement results from the normal and infected RBCs, respectively. In the figure, the conductance, $G=Re\left\{Y\right\}$, is plotted as a function of the point of measurement for each frequency. The frequency values were the same for both normal cells and infected cells, as indicated in Figure 6a. From Figure 6, we can see changes in the conductance (*G*) from the background value in the dotted frames when a cell passes through the electrode gap.

**Figure 7:**
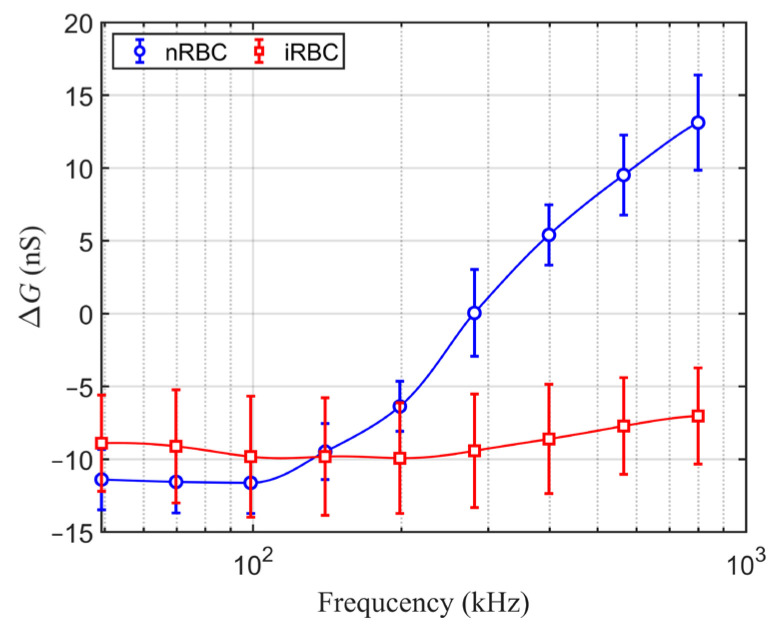
Comparison of conductance changes (Δ*G*) of normal red blood cells and infected red blood cells.

We employed an in-house MATLAB code to estimate the background conductance at the time of each event of the cell. The conductance change (Δ*G*) in relation to the background value was then determined. Figure 7 presents Δ*G* as a function of the electrical frequency (*f*) for the normal cells and the malaria-infected cells. The error bars represent standard deviations. Note that positive Δ*G* means an increase in the conductance between the electrodes, and vice versa. Δ*G* was determined as the average value from the measurement on 48 nRBCs and 80 iRBCs. The figure clearly shows the different Δ*G*-*f* characteristics between the normal cells and the infected cells. For the normal cells, Δ*G* was negative at low frequencies ranging from 50 to 200 kHz and positive at frequencies higher than 400 kHz. Such behavior of Δ*G* agreed with the simulation results in Figure 5a. The critical frequency (*f_c_*) at which Δ*G* changed its sign was at an intermediate frequency between 200 and 400 kHz from the measurement. The *f_c_* value was more or less in the same order as the simulation results, which gave *f_c_* about 100 kHz. The magnitude of the measured Δ*G* varied between −12 and 13 nS, slightly larger than the values obtained from the simulation.

For the malaria-infected red blood cells, it can be seen from Figure 7 that at low frequencies, Δ*G* of the infected cells was higher than that of the normal cells. The difference in Δ*G* characteristics between the normal and the infected cells indicates changes due to parasite invasion in cellular properties, i.e., those of cytoplasm and membrane, as reported in previous research [40,41]. At the low frequencies, Δ*G* is related to the dielectric properties of the cell membrane. Thus, the increase in Δ*G* (reduction of its magnitude) at the low-frequency limit of the infected cells implies a significant increase in cell membrane conductance, which is usually negligible for normal cells. This implication agrees with the results from a recent dielectrophoretic study of malaria-infected red blood cells [6]. However, it should be noted that the statistical significance of the difference in Δ*G* at low frequencies is small, and further investigation is needed to clarify this aspect.

In addition to the increase in Δ*G* at low frequencies for the infected cells, Figure 7 shows that although the Δ*G* for both cell types tended to increase with the frequency for intermediate and high *f* > 300 kHz, the increasing rate was remarkably milder for the infected cells than for the normal cells. At the intermediate and high frequencies, Δ*G* behavior of the infected cells is contributed from both cell membrane capacitance *C_m_* and intracellular conductivity $\sigma_{c}$. Based on the simulation results shown in Figure 5b,c, it was indicated that the infected cells might have smaller *C_m_*, which lowered Δ*G* at intermediate frequencies, and lower $\sigma_{c}$, which reduced Δ*G* at high frequencies of our measurement range. The change in membrane properties might be related to the invasion of the parasite. Upon the invasion, the parasite creates a pore on the membrane and subsequently modifies the physical properties of the membrane such as the formation of knob and the increase in adhesiveness [42].

It is worth noting that the tendencies presented here were distinct from those computed from the impedance measurement results for infected cells in a high-conductivity medium [34]. The difference may be due to the extracellular medium and the double-shell models employed in their computation for the cells. Figure 5c shows that the impedance spectrum depends on the intracellular property to some extent. The use of a double-shell model involves the parasite membrane and interior, which affect the effective intracellular parameters. The change in $\sigma_{c}$ with the conductivity of the extracellular medium was reported [41]. However, with the malaria infection, the change in intracellular electrical properties is complicated by the presence of hemozoin and internal membrane, which should be subject to further study.

For the classification of the infected cells, Figure 7 shows that the difference of Δ*G* values for *f* above 400 kHz was significant between the normal and the infected cells. That is, Δ*G* was positive for normal cells but negative for infected cells. Electrical opacity and impedance phase are often utilized for cell classification [43]. By performing the impedance spectroscopy in a low-conductivity medium, the results obtained here allow a simpler method for the detection of the infected cells based only on the values of Δ*G*. It is worth noting that the detection is based on the change in the membrane properties of red blood cells associated with the parasite invasion. An investigation of electrical characteristics at higher frequencies [44] may be employed to extract changes in intracellular properties due to the presence of hemozoin, which is produced by the parasite in red blood cells. It is also worth noting that an integration with the DEP sorting application can be implemented on a single chip for evaluating the efficiency of the sorting process, which is often performed using a medium of low conductivity.

## 5. Conclusions

This paper presents the electrical impedance spectroscopy of the normal and the malaria-infected red blood cells. The numerical simulation was conducted to estimate the characteristics of the electrical impedance obtained from the microfluidic device and to determine the effect of cell orientation on the impedance spectrum. From the results of the EIS experiments, we focused on the conductance difference Δ*G* from the background value due to the presence of a cell in the sensing electrode gap. The Δ*G*-*f* characteristic of the normal red blood cells agreed with the simulation results. Based on the approximate circuit and the simulation results, the changes in the measured Δ*G* were possibly due to an increase in cell membrane conductance, a decrease in the cell membrane capacitance, and a lowered intracellular conductivity. We have found that by performing the impedance spectroscopy in the low-conductivity medium, the infected cells showed remarkable differences in the Δ*G* characteristic from the normal RBCs, permitting a simple method for the infected cell detection.

## Data Availability

Dataset available on request from the authors.
